# *In silico* improvement of affinity for highly protective anti-malarial antibodies

**DOI:** 10.1016/j.isci.2025.112903

**Published:** 2025-06-14

**Authors:** Mateo Reveiz, Prabhanshu Tripathi, Lais Da Silva Pereira, Patience Kerubo Kiyuka, Tracy Liu, Yongping Yang, Baoshan Zhang, Dorra Benmohamed, Brian G. Bonilla, Carl W. Carruthers, Marlon Dillon, Daniel Gowetski, Sven Kratochvil, Gabriella Lagos, Mariah Lofgren, Ivan Loukinov, Shamika Mathis-Torres, Andrew J. Schaub, Elizabeth Scheideman, Arne Schön, Chen-Hsiang Shen, Yevel Flores-Garcia, Fidel Zavala, Facundo D. Batista, Azza H. Idris, Robert A. Seder, Peter D. Kwong, Reda Rawi

**Affiliations:** 1 Vaccine Research Center, National Institute of Allergy and Infectious Diseases, National Institutes of Health, Bethesda, MD, USA; 2 Department of Biology, Johns Hopkins University, Baltimore, MD, USA; 3 The Ragon Institute of Massachusetts General Hospital, Massachusetts Institute of Technology and Harvard University, Cambridge, MA, USA; 4 Department of Immunology, Harvard Medical School, Boston, MA, USA; 5 Department of Microbiology, Harvard Medical School, Boston, MA, USA; 6 Malaria Research Institute, Johns Hopkins Bloomberg School of Public Health, Baltimore, MD 21205, USA; 7 Aaron Diamond AIDS Research Center, Columbia University Vagelos College of Physicians and Surgeons, New York, NY 10032, USA; 8 Department of Biochemistry and Molecular Biophysics, Columbia University, New York, NY 10032, USA; 9 Kenya Medical Research Institute, Centre for Geographic Medicine Research Coast, P.O. Box 230-80108, Kilifi, Kenya; 10 Department of Biology, Massachusetts Institute of Technology, Cambridge, MA, USA

**Keywords:** Biological sciences, Immunology, Natural sciences, Structural biology

## Abstract

The monoclonal antibody CIS43 preferentially binds the junctional region of *Plasmodium falciparum* circumsporozoite protein (PfCSP) and is highly protective in humans. Here, we develop an *in silico* pipeline to improve antigen-antibody interaction energies and apply it to CIS43 variants elicited in CIS43-germline knock-in mice. Improved binding of CIS43 variants to the CIS43 junctional epitope (PfCSP peptide 21) was achieved by introducing single and double amino acid substitutions in the peptide 21-proximal heavy- and light-chain-variable regions. The best *in silico* designed variant, antibody P3-43-LS, was 2- to 3-fold more protective than antibody CIS43-LS, the clinical version of CIS43 with half-life extending leucine-serine (LS) mutations, and had comparable protection to the current best-in-class antibody (iGL-CIS43.D3-LS) to this region. Crystal structures of the improved antibodies revealed atomic-level interactions accounting for gains in binding affinity. This *in silico* approach to improve antibody affinity can thus be used to enhance potency of PfCSP monoclonal antibodies.

## Highlights

• An *in silico* pipeline was developed to augment antibody-antigen binding affinities
• Designed antibodies have enhanced affinity for peptide 21 of circumsporozoite protein
• Functional analyses reveal protective efficacy of variants to surpass antibody CIS43
• Crystal structures elucidate interactions responsible for increased affinities

## Graphical abstract

**Figure:**
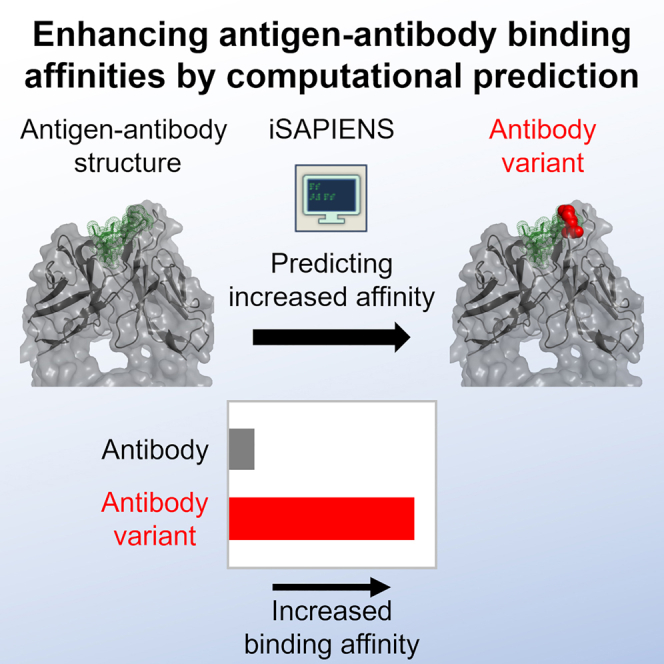

## Introduction

Malaria is an infectious disease caused by several species of *Plasmodium* parasites. In 2020, there were 241 million cases of malaria and 627 thousand deaths worldwide, with children under 5 accounting for an estimated 80% of all malaria-related deaths in the African region.^1^ *P. falciparum* is the most prevalent, accounting for ∼90% of mortality cases.^2^ Infection occurs when an infected female *Anopheles* mosquito bites a human and injects sporozoites, which rapidly migrate to the bloodstream. These quickly invade hepatocyte cells in the liver, where they reproduce for ∼7 days before releasing thousands of merozoites to the bloodstream, starting the erythrocytic stage leading to clinical symptoms.^3^

The *P. falciparum* circumsporozoite protein (PfCSP) is the predominant surface antigen of sporozoites^4^ as well as the primary target of vaccines such as RTS,S and R21 which display a number of NANP repeats as well as the C-terminal region that is presented as a particle.^5^^,^^6^^,^^7^ PfCSP is also the target for multiple human monoclonal antibodies, such as CIS43, MGG4, and L9 (elicited from the attenuated PfSPZ vaccine); 311 and 317 (elicited from the RTS,S vaccine); and 580g or 663 (from natural exposure).^8^^,^^9^^,^^10^^,^^11^^,^^12^ CIS43 preferentially targets the junctional region referred to as peptide 21 (residues 101–115 of PfCSP). The Fc-modified variant, CIS43-LS was developed to increase the half-life *in vivo* and was shown to be highly protective in a phase I human clinical trial following a single administration against controlled human malaria infection or intense seasonal infection over 6 months in Mali.^10^^,^^13^^,^^14^ These studies provided proof of principle that a single infusion of monoclonal antibodies can prevent malaria over the course of an entire rainy season. To further enhance the potency of CIS43-type antibodies, two variants m42.127 and m43.151 were elicited from a CIS43-germline knock-in mice following vaccination with peptide 21 linked to keyhole limpet hemocyanin (KLH). Both of these antibodies showed better protection than CIS43 in a mouse model of malaria infection.^15^ These antibodies were then used to design the best-in-class antibody, iGL-CIS43.D3 (D3).^15^ These results demonstrated that using CIS43-germline knock-in mice model can improve the potency of antibodies. However, it will be critical to further find approaches to increase the potency of monoclonal antibodies against malaria to allow for lower dosing and reduced costs.

Antibody enhancement can be achieved through various techniques, encompassing both experimental and computational strategies. The growing computational capabilities underscore the promise of *in silico* methodologies. While direct *in silico* predictions of liver burden protection are currently unattainable, it is possible to employ the interaction energies between *in silico* mutated antibody variants and the junctional peptide 21 as a surrogate measure for binding affinity. Previously, we demonstrated that the binding affinity shows a relationship with improved liver burden protection.^15^ Due to the large number of potential antibody mutations to be screened, we devised a scalable methodology capable of accommodating thousands of variants. This approach involves the mutation of specific residues, coupled with brief energy minimization processes, and is subsequently followed by a singular molecular dynamics (MD) simulation to compute the interaction energies related to interface and stability. Our objective was to apply this methodology to two antibodies, m42.127 and m43.151, both of which are well characterized for their high potency and possess available crystal structures. The resulting mutant antibodies and their corresponding interaction energies were selected as pareto optimal solutions for experimental antigenic assessment by AlphaLISA and biolayer interferometry (BLI) as well as for functional *in vivo* assessment in the mouse malaria challenge model. To further understand the atomic-level interactions leading to higher binding affinity, we performed residue pairwise energy analysis of the resulting *in silico* models and compared these results experimentally by determining crystal structures of top variant antibodies in complex with peptide 21. Overall, our results demonstrate the ability of the *in silico* pipeline to improve antibody affinities to its antigen, with the best obtained antibody, P3-43, showing higher binding compared to its template antibody m43.151. Additionally, we showed that P3-43 has higher protection than its ancestral antibody CIS43 and comparable *in vivo* protection to the half-life extended version of D3 (D3LS), the current best-in-class antibody for junctional antibodies.

## Results

### *In silico* antibody improvement pipeline

**Figure 1:**
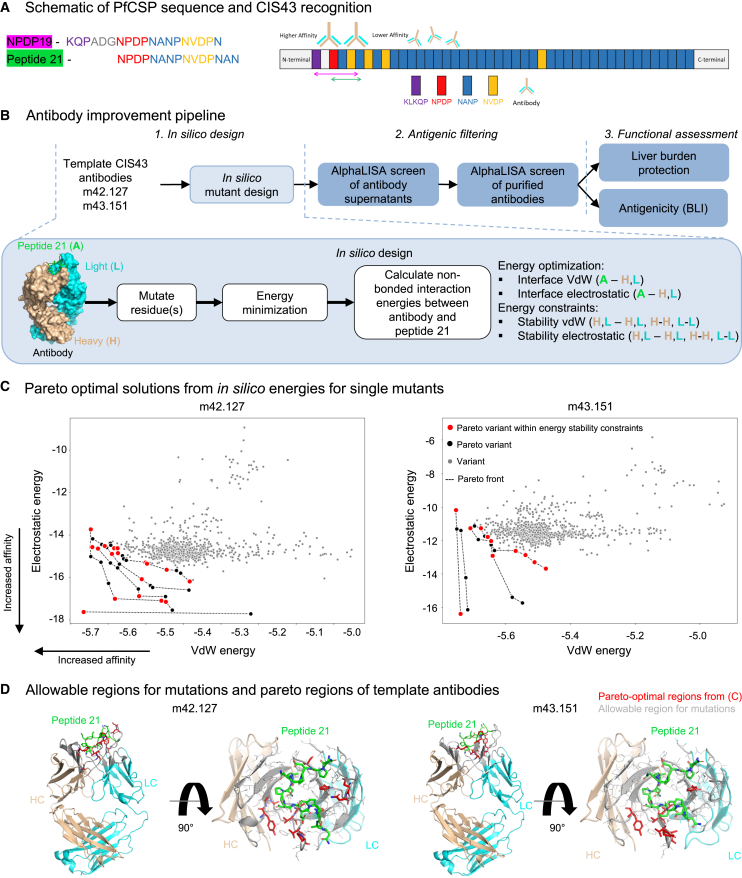
Antibody improvement pipeline identifies CIS43 variants with improved *in silico* non-bonded interaction energies to junctional peptide 21 (A) Schematic of PfCSP sequence and junctional peptides. (B) Antibody improvement pipeline is composed of three sequential steps. First, *in silico* interaction energies are calculated. Second, antigenic filtering down-selects most promising antibody variants. Lastly, functional assessment compares antibody variants against current best-in-class antibodies. (C) Pareto optimal solutions from *in silico* energies for all single mutants based on m42.127 and m43.151 templates. Mutations within the top pareto fronts are depicted in black and red circles. Variants satisfying all stability constraints are highlighted in red. (D) Allowable region for mutations within 12 Å of peptide 21 (green) for m42.127 (left) and m43.151 (right) is shown in gray. Residue positions with pareto-optimal energies identified *in silico* are shown in red. See also Figures S1 and S3.

We established a computational framework aimed at enhancing the binding affinity of CIS43 antibody variants through the optimization of interaction energies with the PfCSP-junctional peptide 21 (Figure 1A). Beginning with the published crystal structures of the two template antibodies, m42.127 and m43.151, which are complexed with peptide 21 (PDB IDs: 7LKB and 7LKG), we generated all possible single amino acid substitutions in the heavy (VH) and light (VL) variable regions located within 12 Å of the peptide (Figure 1D). For each mutant, we performed a short energy minimization to remove side-chain clashes using FoldX and YASARA software.^16^ A 2-dimensional interface energy space consisting of non-bonded van der Waals (vdW) and electrostatic interaction energies between the peptide and the antibody residues within 12 Å of the peptide were calculated (interface metrics) using NAMD. The heavy to heavy (H-H), light to light (L-L), and heavy to light (H-L) chain interaction energies were also calculated and treated as stability measures (Figure 1B; Table S1). The best single mutants were first selected by taking the top pareto fronts in the 2-dimensional interface energy space. The stability measures in the remaining 6-dimensional space were simply used as ε-constraints as is commonly done in multi-objective optimizations; that is, that mutants with larger stability energies than ε = 1 standard deviation from the average energy were discarded. For the m42.127 template, 42 mutants were found in the top five pareto fronts of which 19 satisfied the ε-constraints (Figure 1C left). For the m43.151 template, 23 mutants were found in the top four pareto fronts while 11 were within the allowed stability regions (Figure 1C right). The top single mutants were subsequently used to generate double mutants by following a greedy combinatorial approach. From the 19 m42.127-based single variants, $\left(\begin{array}{c} 19\\ 2 \end{array}\right)=171$ double combinations were assessed and down-selected by using the same pareto-based approach described for the single variants. Four final variants of the first pareto front within stability bounds were selected. From the 11 m43.151-based single variants, 55 combinations were assessed for a final of 6 variants (Figure S1). Overall, we down-selected a total of 29 single and 6 double mutants (35 total variants) for experimental antigenic assessment.

### Antigenic characterization confirms increased affinity to junctional peptide 21

**Figure 2:**
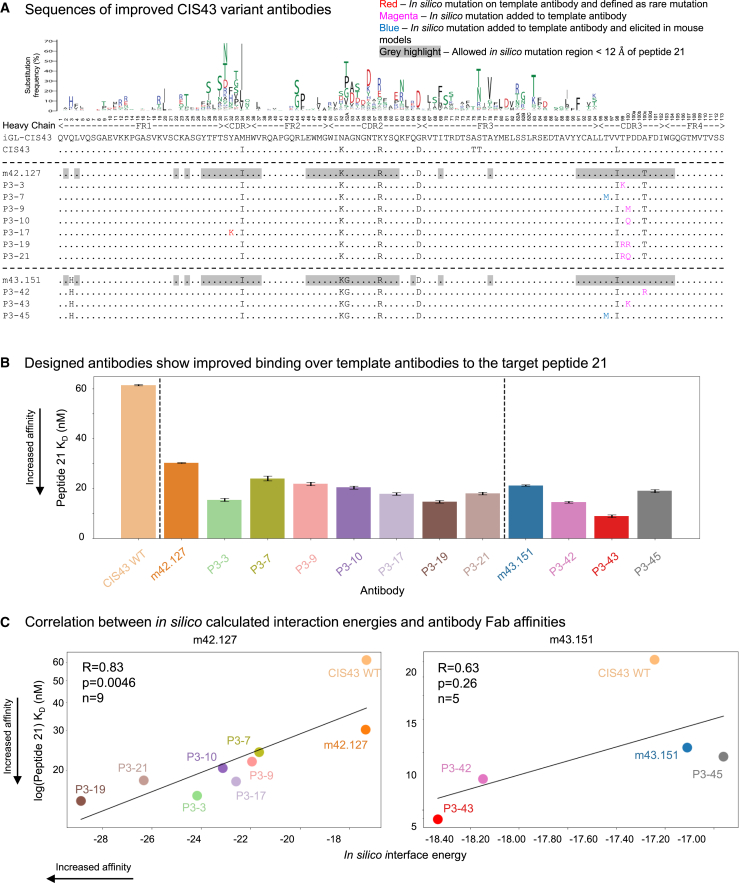
*In silico* designed CIS43 variants show improved binding to peptide 21 (A) Heavy chain sequences for the top mutants as characterized by purified AlphaLISA signals. (B) Biolayer interferometry (BLI) affinity of selected CIS43 variants to peptide 21. Variants are grouped depending on their template antibody. Error bars correspond to the standard error in affinity measurement. (C) *In silico* interface energy strongly correlates to peptide 21 BLI Kd measurements for m42.127-based variants. See also Figure S2.

The 35 *in silico* designed antibody variants were produced and antigenically assessed using AlphaLISA. First, the supernatants of the antibodies were expressed and assessed for their reactivity to the junctional peptide NPDP19, as the affinity to NPDP19 has been shown to have high correlation with function and malaria protection efficacy.^15^ From the initial 35 *in silico* designs, 27 successfully expressed (Table S2) and 11 showed improved binding when compared to m43.151 (Figure S2A). Subsequently, the 11 top antibody variants were purified and re-assessed against peptide 21 using AlphaLISA at two different IgG concentration, in particular 1 nm (Figure S2B) and 10 nm (Figure S2C) to avoid hook effects and to allow a more expansive selection of antibodies. Out of the 11 selected antibodies, only antibody P3-44 containing the mutation P100R_H_ did not show improvement over its template antibody m43.151 and was not further considered (Figures S2B and S2C). The selected antibodies based on the m42.127 template include five single variants with mutations in the CDRH3 region (T99K_H_, T96M_H_, P100M_H_, and P100Q_H_) and one rare mutation in the CDRH1 region (Y32K_H_) (Figure 2A). Two multi-mutants were also selected with CDRH3 mutations (T99R_H__P100R_H_ and T99R_H__P100Q_H_). Based on the m43.151 template, four single variants are concentrated in the CDRH3 region (A100cR_H_, P100K_H_, P100R_H_, and T96M_H_). Overall, predictions suggest position 100 in the heavy chain to be important as it was frequently mutated. Most of the mutations identified by the *in silico* pipeline had not been elicited in previous murine models from which the template antibodies where derived (days 13 and 28 from immunization), with the exception of T96M_H_ (Figure S1B).^15^

Next, we produced the antibody-binding fragments (Fabs) of the antibodies and measured binding affinities to the target peptide 21 using BLI, as well as to NPDP19 and PfCSPm (Figure 2B; Figures S3A and S5). Binding to peptide 21 was improved for all selected antibodies by ∼2-fold on average with respect to their template antibodies m42.127 (30.2 ± 0.2 nM) and m43.151 (21.2 ± 0.2 nM), with P3-43 showing the highest binding with 8.9 ± 0.5 nM (Figure 2B). Interestingly, the calculated interaction energies showed strong significant correlation with the experimentally determined binding affinities after log normalization as expected from the Boltzmann equation (m42.127: R = 0.83, *p* value = 0.0046; m43.151: R = 0.63, *p* value = 0.26 with only five measurements) validating the *in silico* approach for the m42.127 antibody (Figure 2C).

### Functional characterization of designed variants reveals new best-in-class antibody

**Figure 3:**
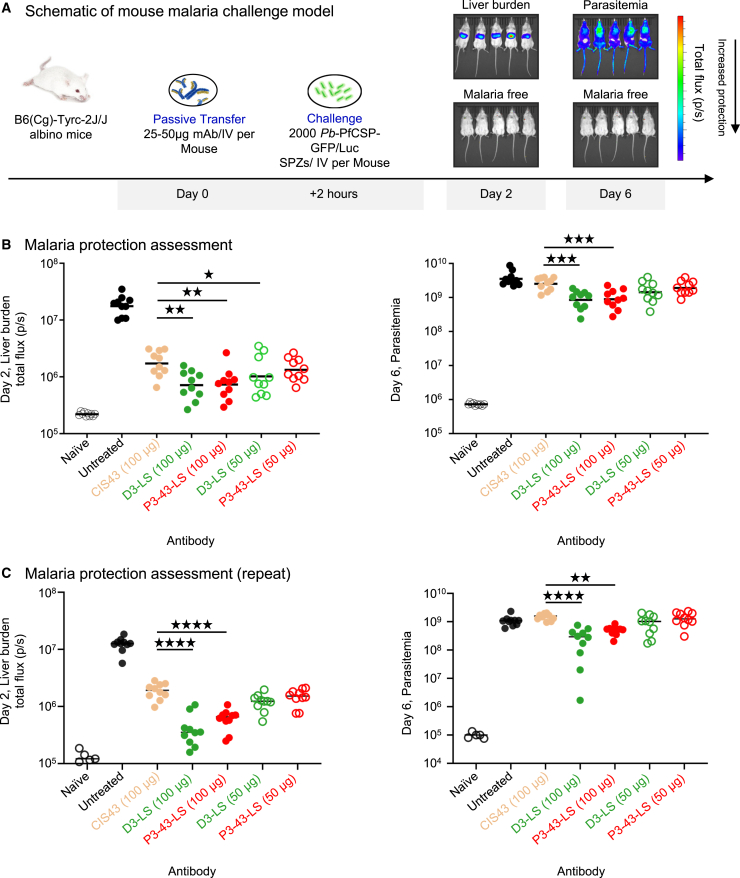
Functional characterization reveals best *in silico*-designed antibody P3-43 is superior to clinical template antibody CIS43 (A) Schematic of mouse malaria challenge model. (B) Liver burden protection and parasitemia at 50 μg and 100 μg dosage were assessed for iGL-CIS43.D3-LS and P3-43-LS along with clinical antibody CIS43-LS at dosage of 100 μg. Relative to clinical antibody CIS43-LS, both iGL-CIS43.D3-LS and P3-43-LS are significantly more protective as assessed by uncorrected Dunn test. (C) Repeat protection experiments, conducted by a different research team, showed an almost identical outcome with iGL-CIS43.D3-LS and P3-43-LS being significantly more superior than CIS43-LS, as assessed by uncorrected Dunn test.

We hypothesized that improved junctional peptide affinity would result in antibodies with higher protection *in vivo*. To evaluate the potency of these variants, we assessed their ability to limit malaria infection in an *in vivo* murine challenge model.^12^ To directly compare the variant antibodies generated to the parent clinical antibody CIS43-LS, and the current best-in-class antibody D3-LS, we expressed all antibodies incorporating the half-life extending Fc mutation LS. The LS mutation has no effect on potency compared to the wild-type antibody.^17^ Following passive transfer of antibody, mice were challenged with *Plasmodium berghei* (Pb) parasites expressing PfCSP and a green fluorescent protein/luciferase fusion protein (Pb-PfCSP-GFP/LUC SPZ). Thereafter, parasite burden was detected by measuring whole-body bioluminescence and the results are expressed as the reduction in liver burden (Figure 3A). Relative to the parental CIS43-LS antibody, mice that received P3-43-LS or D3-LS at a dose of 100 μg showed significantly higher protection using the uncorrected Dunn’s test as reflected in reduced liver burden, measured at day 2 post-infection, and parasitemia, measured at day 6 post-infection (∼2- to 3-fold improvement in raw signal) (Figure 3B). To ensure reproducibility of our initial findings, we conducted the same experiment in a different laboratory and obtained almost similar results (Figure 3C).

### *In silico* energies and interatomic interactions provide structural insight

**Figure 4:**
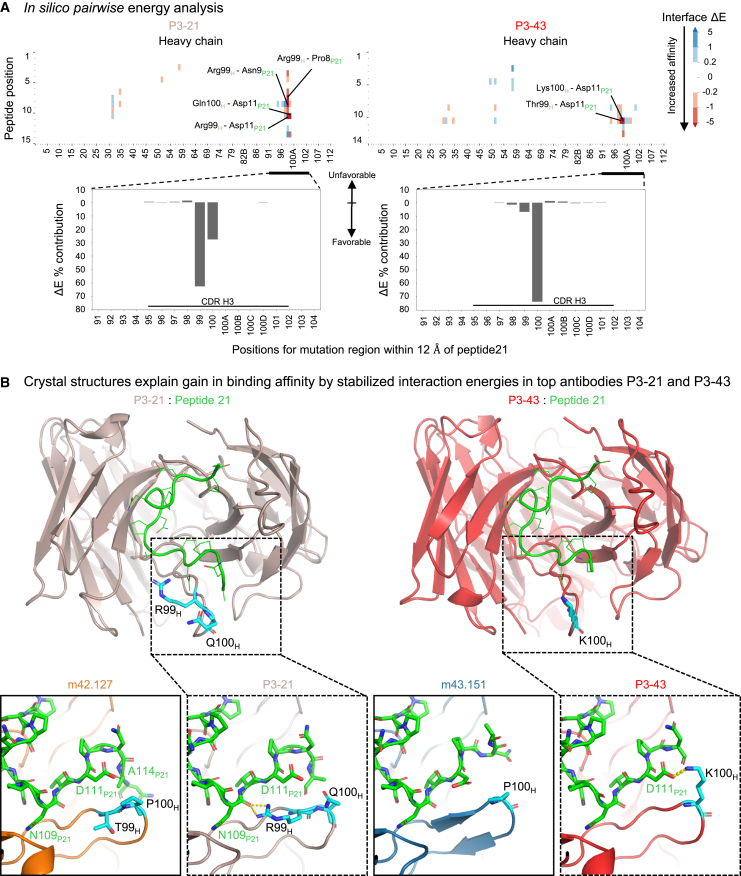
*In silico* energies and crystal structure of P3-21 in complex with peptide 21 depict additional atomic interactions explaining increase in affinity (A) *In silico* pairwise energy analysis. For each pair of residues between peptide 21 (*y* axis) and the antibody (*x* axis), the total interface energy for the antibody is subtracted from the corresponding template antibody. Lower values indicate more favorable interaction energies. In the bar plots below, values are summed across the peptide 21 positions to further examine the CDRH3 region. (B) Crystal structures of P3-21 (top left panel in brown) and P3-43 (top right panel in red) in complex with peptide21 (green) is shown in cartoon illustration. Mutated residues are depicted in cyan stick conformation. Critical binding regions (bottom dotted panels), including heavy chain position 99 and 100 of template antibody m42.127 and P3-21 in complex with peptide 21. Amino acid Arg99 in P3-21 introduces new electrostatic interactions with peptide 21 backbone atoms. Interatomic interactions from residue 99 in heavy chain and residue 9 in peptide 21 is displayed and compared with that of template m42.127 crystal structure. P3-43 mutation Lys100 introduces new interactions with peptide 21 Asp11. See also Figure S4.

To understand further the impact of the introduced mutations, we first generated a pairwise interaction energy matrix between peptide 21 and all antibody residues. We then calculated ΔE by subtracting the variant matrix by its corresponding template antibody matrix. For one of the best m42.127 based variants *in vivo*, P3-21, most of the interaction energy gain originates from the introduced residues Arg99_H_ and Gln100_H_, both located in the CDRH3. Their interaction energies account for 63% and 28% for Arg99 and Gln100, respectively (Figure 4A bottom left). Interestingly, both Arg99_H_ and Gln100_H_ residues strongly interact with peptide 21 residue Asp11, with Arg99 additionally showing strong interactions to peptide 21 residues Pro8 and Asn9 (Figure 4A top left). Regarding the best m43.151 based variant P3-43, the majority of interaction energy gain originates from the introduced residue Lys100_H_ in the CDRH3 with 74% of overall energy contribution (Figure 4A bottom right). The energy gain is mostly originating from interactions with peptide residues Asp11 and Ala14 (Figure 4A top right). Also, P3-42 shows a similar trend where newly introduced positively charged residue Arg100c_H_ moderately improves the electrostatic energy with negatively charged peptide 21 Asp11 and vdW energy with Asn13 (Figure S4A top). In general, the gain in interaction energy originates from electrostatic rather than vdW improvements.

### Structural analysis reveal interactions leading to increased affinity to junctional peptide 21

To analyze the atomic-level interactions leading to improved binding, we sought to determine the crystal structures of the top antibodies. We co-crystallized antibody variants P3-21, P3-42, and P3-43 with peptide 21 and determined their structures in complex with peptide 21 to 2.2 Å (Figure 4B left panel; Table S3), 1.8 Å (Figure S4B), and 2.3 Å resolution, respectively (Figure 4B right panel; Table S3). Overall, the modeled and experimentally determined structures show low backbone deviations within their CDR loops with an average backbone root-mean-square deviation (RMSD) of 0.52 Å, 0.88 Å, and 0.72 Å for P3-21, P3-42, and P3-43, respectively.

The analysis of P3-21 co-complex structure showed additional interactions between the introduced mutations and peptide 21. In particular, the Thr99Arg_H_ mutation allowed the side chain of Arg99_H_ to interact with peptide 21 via hydrogen bonding interactions with the backbone carbonyl of Asn109_P21_ (depicted as yellow dashed lines in Figure 4B left panel). The introduction of Pro100Gln_H_ mutation is thought to increase flexibility of the CDR-H3 loop, resulting in a weak hydrogen bond between Gln100_H_ and Asp111_P21_. P3-1 variant (m42.127_Thr99Arg_H_) that lacks the Pro100Gln_H_ did not show improved apparent affinity to peptide 21 in the AlphaLISA measurements. P3-43 showed the highest affinity to peptide 21 (Figure 2B) and the structural analysis of P3-43 in complex with peptide 21 revealed a strong salt bridge interaction between the introduced Pro100Lys_H_ mutation and Asp111_P21_, as predicted by the *in silico* model (74% contribution by Lys100_H_ predicted) (Figure 4B right panel). The additional interaction suggested by the *in silico* model between Thr99_H_ and peptide 21 Asp11 was however not visible on the crystal structure as the side chain of Thr99_H_ is facing outwards, while the short energy minimization during the *in silico* MD step led to the Thr99_H_ facing toward the Asp11 residue.

For the crystal structure of antibody P3-42, the introduced Arg100c_H_ residue is substantially distant from Asn109_P21_ (Figure S4B). Long-range electrostatic interaction energies may provide a basis for the observed increase in binding affinity, particularly between Asp111_P21_ and Arg100c_H_ as suggested by the *in silico* model.

Overall, the *in silico* model pairwise analysis revealed the atomic-level interactions contributing to the increase in affinity as shown by the corresponding crystal structures.

## Discussion

In this study, we show that an *in silico* energy-based pipeline is able to identify key mutations that improve binding to the junctional peptide 21 as confirmed by antigenic assessment using BLI. The binding improvement observed in this study with the variant P3-43 demonstrated a significant decrease in liver burden, achieving an approximate 2– to 3-fold improvement compared to the benchmark CIS43 antibody, which has previously exhibited high-level efficacy in clinical trials. The protection observed in the mouse model cannot be solely ascribed to improved binding; instead, it is shaped by a complex interplay of various biological factors. For example, PfCSP’s extended spiral conformation of the repeat regions allows for complex inter-Fab contacts; mutations to non-antigen positions could enhance or decrease the potency of antibodies.^18^ In general, the correlation between affinity and functional improvement may greatly vary depending on the biological system studied which can change the functional success rate of this method. Multiple studies have showed PfCSP-binding affinity correlations with functional protective capacity, in particular the dissociation rate (k_off_), though other studies have shown more limited correlations at higher affinity, hence, posing affinity as a required but insufficient condition to high inhibitory capacity.^19^^,^^20^

While we were able to improve affinity over the best natural antibodies m42.127 and m43.151, these antibodies were already highly optimized and contained 6 and 7 mutations in the heavy chain, respectively, with respect to iGL-CIS43. From a methodological development point of view, it would have been interesting to investigate the performance of the algorithm when starting from germline iGL-CIS43 or WT-CIS43 mature variants for a more direct comparison to the mice models used to discover the template antibodies. It is plausible that antibodies similar to m42.127 and m43.151 may be identified with only a limited number of mutations. However, in this work, we sought to directly optimize the top murine antibodies to rapidly discover new potential clinical products. We recognize that while multiple consecutive rounds of the *in silico* method could provide valuable information, large deviations from the template antibody are unlikely to provide accurate *in silico* energy values due to the short nature of the MD step and restricted backbone movement in our pipeline. Nonetheless, this *in silico* approach enables sampling of all potential amino acid substitutions, which is not possible in an *in vivo* system, due to other biological factors and constraints. Also, our pipeline was set up to efficiently sample many different mutations, with the caveat that we could not perform extensive conformation sampling of the introduced mutants, for instance using MD. Hence, we did not account for diverse backbone conformations after introducing large residues, which might affect overall interaction energy contributions.

While this method was not designed to generate *de novo* antibodies and requires an *a priori* binding antibody in complex with its target, we proved that it can be effectively used to refine and optimize antibody variants when used in conjunction with *in vivo* models. Interaction energies for one single variant can be calculated in ∼5 min using 24 CPUs. Simulating all peptide-proximal mutations using high-performance computing settings can be accomplished within days, while smaller computational platforms might require more time. This *in silico* pipeline provides a powerful and generally applicable approach to improve antibody functionality and leads in this case to the design of antibodies to be used for passive prevention against malaria, by further optimizing CIS43 variants not only against different epitopes such as peptide 29 or additional antibodies, such as L9, but also to additional infectious disease targets.

### Limitations of the study

While our *in silico* pipeline successfully optimized antibody binding, several limitations remain. The correlation between binding affinity and functional protection is complex, and better affinity does not always correlate with improved protection.^19^^,^^20^ Additionally, we note that *in vitro* assays do not reliably predict the functional capacity of antibodies *in vivo*. Consequently, we note that the relative potency of the monoclonal antibodies is determined in the murine malaria challenge model that utilizes a transgenic *P. berghei*, which expresses *PfCSP*, to create a framework for the comparative analysis of monoclonal antibodies. This model, however, is not without its limitations, as it employs *P. berghei* rather than *P. falciparum* sporozoites. Phase 1 trials demonstrated that 23 μg/mL of CIS43-LS and 10 μg/mL of L9LS were sufficient to protect humans from five PfSPZ-infected mosquito bites, whereas much higher titers—364 μg/mL and 145 μg/mL, respectively—were needed to achieve 80% protection in mice against Pb-PfCSP-SPZ-infected bites. This discrepancy indicates that mice in the *P. berghei* model require higher PfCSP antibody titers for protection, highlighting the need for ongoing post-hoc analyses to assess the predictive accuracy of murine models for monoclonal antibody potency in humans.

## Resource availability

### Lead contact

Further information should be directed to and will be fulfilled by Reda Rawi (reda.rawi@nih.gov).

### Materials availability

Requests for resources and reagents should be directed to and will be fulfilled by Reda Rawi (reda.rawi@nih.gov). All new reagents are available by MTA for non-commercial research.

### Data and code availability

#### Data

*In silico* energies and sequence information required to run the computational pipeline for selected antibody variants can be found in Table S1. Variants summary and data availability of antibodies can be found in Table S2. Resolved structures have been added to the PDB: 7UFQ, 7UFN, and 7UFO. X-ray crystallography data collection and refinement statistics can be found in Table S3.

#### Code

iSAPIENS source code is publicly available as of the date of publication under https://github.com/RedaRawi/iSAPIENS. Instructions on how to run the pipeline are available on the GitHub repository.

#### Other items

Any additional information required to reanalyze the data reported in this paper is available from the lead contact upon request.

## Acknowledgments

We thank J. Gall for assistance with antibody production, J. Stuckey for assistance with figures, and members of the Structural Biology Section and Structural Bioinformatics Core, Vaccine Research Center, for discussions and comments on the manuscript. Support for this work was provided by the 10.13039/100016820Vaccine Research Center, an intramural Division of National Institute of Allergy and Infectious Diseases, NIH, by 10.13039/100000002NIH 10.13039/100000060NIAID R01 AI168114 and R01 AI151178, and by Leidos Biomedical Research Inc. No. 20X013F. This work utilized the computational resources of the NIH HPC Biowulf cluster (http://hpc.nih.gov). Use of sector 22 (Southeast Region Collaborative Access team) at the Advanced Photon Source was supported by the US Department of Energy, Basic Energy Sciences, Office of Science, under contract number W-31-109-Eng-38.

## Author contributions

M.R. and R.R. headed the development of the *in silico* pipeline, performed computational calculations, and led the study. P.T. purified antibodies, performed BLI and AlphaLISA experiments, made Fabs, and determined the crystal structures. B.Z. and T.L. performed large scale transfection of antibodies. Y.Y. performed 96-well plate transfections. L.D.S.P., P.K.K., B.G.B., M.D., and M.L. assessed malaria protective efficacy of antibodies in mouse models. C.-H.S. performed sequence analysis. A.S. performed ITC experiments. D.B., C.W.C., D.G., G.L., I.L., and E.S. produced D3 and P3-43 using CHO stable pools. S.M.-T., Y.F.-G., and F.Z. co-led D3 and P3-43 protective efficacy measurements. S.K. and F.D.B. provided template antibodies m42.127 and m43.151. A.H.I. co-led variant PfCSP antibody protective efficacy measurements. R.A.S. oversaw malaria protection studies. A.J.S. computed BSA metrics and rendered structure figures. P.D.K. and R.R. oversaw the project. M.R. and R.R. provided the main draft of the manuscript, to which all authors provided revisions or comments.

## Declaration of interests

S.K., P.T., R.R., M.R., P.D.K., R.A.S. and F.D.B. have submitted a US Provisional Patent Application describing improved CIS43 antibodies (filed November 5, 2021). R.A.S. and A.H.I. hold patents on CIS43 (International Application No. PCT/US2018/017826; US patent application no. 16/485,354; issued June 1, 2021). F.D.B. has consultancy relationships with Adimab, Third Rock Ventures, and the EMBO Journal, and founded BliNK Therapeutics.

## STAR★Methods

### Key resources table

**Table:** 

REAGENT or RESOURCE	SOURCE	IDENTIFIER
**Chemicals, peptides, and recombinant proteins**		

Recombinant PfCSP	Produced in house	N/A
Biotinylated junctional peptide Pep21	Genscript	Order ID:# U134AFB120
Biotinylated junctional peptide NPDP19	Genscript	Order ID:# U134AFB120

**Critical commercial assays**		

Streptavidin Donor Beads, for AlphaLISA	PerkinElmer	CAT#:6760002
Anti-human Fc Acceptor Beads, for AlphaLISA	PerkinElmer	CAT#:AL103M
Octet® Streptavidin (SA) Biosensor	Sartorius	CAT#:18-5019

**Deposited data**		

PDB file	This paper	7UFQ
PDB file	This paper	7UFN
PDB file	This paper	7UFO

**Experimental models: Cell lines**		

Human: Expi293 cell	Thermo Fisher Scientific	Cat#A14527

**Experimental models: Organisms/strains**		

Mouse: B6(Cg)-Tyrc-2J/J albino	The Jackson Laboratory	JAX:000058
Mouse: Balb/c	The Jackson Laboratory	JAX:000651
Sporozoite: *P. berghei* sporozoite expressing PfCSP, GFP, and luciferase	Flores-Garcia et al.^21^	N/A

**Recombinant DNA**		

*Plasmodium falciparum* circumsporozoite protein (clone 3D7)	PlasmoDB	PF3D7_0304600.1
pVRC8400 huIgG1	Genscript	N/A
pVRC8400 huIgK	Genscript	N/A

**Software and algorithms**		

Prism 9.0.1	GraphPad	https://www.graphpad.com/
Microsoft Office	Microsoft	https://www.office.com/
IMGT/V-quest	Brochet et al.^22^	http://www.imgt.org/IMGTindex/V-QUEST.php
Geneious 2020.2	Biomatters	https://www.geneious.com
Coot v0.9.4	Emsley et al.^23^	https://www2.mrc-lmb.cam.ac.uk/personal/pemsley/coot/
ChimeraX v1.2.5	Goddard et al.^24^	https://www.cgl.ucsf.edu/chimerax/docs/user/index.html
Phenix v1.18.2	Adams et al.^25^	https://www.ks.uiuc.edu/Development/Download/download.cgi?PackageName=NAMD
The PyMOL Molecular Graphics System	Schrödinger, LLC	https://pymol.org/2/
Gene-specific substitution profile	Sheng et al.^26^	https://cab-rep.c2b2.columbia.edu/
YASARA	Krieger & Vriend^27^	http://www.yasara.org
R	N/A	https://www.r-project.org/
FoldX	Schymkowitz et al.^16^	https://foldxsuite.crg.eu/
VMD	Humphrey et al.^28^	https://www.ks.uiuc.edu/Research/vmd/
NAMD	Phillips et al.^29^	https://www.ks.uiuc.edu/Research/namd/
Python	N/A	https://www.python.org/
iSAPIENS	This paper	https://github.com/RedaRawi/iSAPIENS

**Other**		

IVIS® Spectrum *In Vivo* Imaging System	PerkinElmer	N/A
SpectraMax® i3x	Molecular Devices	N/A

### Experimental model and study participant details

#### Murine malaria challenge model

Female B6(Cg)-Tyrc-2J/J albino (B6-albino) and BALB/c mice, at 6- to 8- week-old, were purchased from The Jackson Laboratory. All mice were maintained in facilities accredited by the American Association for Accreditation of Laboratory Animal Care and cared for according to their standards. All procedures involving animals were performed according to National Institute of Allergy and Infectious Diseases (NIAID), National Institutes of Health (NIH) guidelines for use and care of live animals approved by the Institutional Animal Care and Use Committees (Animal Study Protocols VRC-17-702 and VRC-20-0855).

#### Sporozoite and mosquito malaria challenge model

Transgenic *Plasmodium berghei* (strain ANKA 676m1C11, MRA-868) expressing full-length PfCSP (3D7 strain) full-length *P. falciparum* CSP and a green fluorescent protein/luciferase fusion protein and a GFP/luciferase fusion protein (Pb-PfCSP-GFP/LUC SPZ) were propagated and used to evaluate the efficacy of the PfCSP-directed mAbs, as previously described.^21^ Briefly, BALB/c mice were infected with Pb-PfCSP-GFP/LUC SPZ-infected RBCs. Female *Anopheles stephensi* (Nijmegan) mosquitoes, reared at the Laboratory of Malaria and Vector Research (NIAID, NIH), were allowed to feed on the parasitized BALB/c mice. Blood-fed mosquitoes were then maintained in a humidified incubator at 19°C–20°C and supplied with 10% sucrose. Eighteen to 21 days after an infectious blood meal, sporozoites were harvested from mosquito salivary glands for mouse challenge studies.

### Method details

#### *In silico* interaction energy calculations

Starting with the template pdb files for m42.127 and m42.151 in complex with peptide 21, we first calculated the minimum distance between any antibody residue atom and any peptide atom. Residues within 12 Å were selected and allowed to mutate using the BuildModel command from FoldX software (http://foldxsuite.crg.eu/). The variants were energy minimized using YASARA (http://www.yasara.org/). The reference structures were also modeled and energy minimized by making V1V_H_ and Q1Q_H_ identity mapping mutations. A single molecular dynamics frame was run using the NAMD force field. The energies computed cover both electrostatic and vdW interface energies F(x) (heavy/light to peptide pairs) and stability energies G(x) (heavy to heavy, light to light and heavy to light pairs). Energies from both capped C and N termini were ignored to avoid artifacts and more closely match their native state in the PfCSP protein. Energies were normalized by the number of residues in peptide 21 (15 residues for m42.127 crystal structure and 14 for m43.151). After computing the interaction energies for all single mutants X, the problem becomes to down-select the best performing variants by minimizing the multi-objective function F(x) while constraining the stability function G(x). To do this, the pareto fronts for the two interface energies were calculated using custom python scripts. A pareto front here is defined as the set of all pareto optimal solutions and in this context, a mutant u is a member of the pareto front if:

$$f_{i}\left(u\right)\leq f_{i}\left(v\right)\;\forall \;i\in \left\{1,2\right\}\;\text{and}\;\exists j\in \left\{1,2\right\}:f_{j}\left(u\right)<f_{j}\left(v\right)$$

$$\text{Where}\;F\left(x\right)=\left[\begin{array}{c} f_{1}\left(x\right)=VdW\left(x,HL\right)\\ f_{2}\left(x\right)=Electrostatic\left(x,HL\right) \end{array}\right]$$

$$G\left(x\right)=\left[\begin{array}{c} g_{1}\left(x\right)=VdW\left(H,H\right)\\ g_{2}\left(x\right)=Electrostatic\left(H,H\right)\\ g_{3}\left(x\right)=VdW\left(L,L\right)\\ g_{4}\left(x\right)=Electrostatic\left(L,L\right)\\ g_{5}\left(x\right)=VdW\left(HL,HL\right)\\ g_{6}\left(x\right)=Electrostatic\left(HL,HL\right) \end{array}\right]$$

u,v,x∈X

The standard deviation plus the mean of the remaining six stability energies were calculated and used and constraints to the G(u) stability function, such that variant u would be included if $g_{i}\left(u\right)<\;\mu_{i}+\sigma_{i}\text{.}$ Here, $\mu_{i}$ is the average of $g_{i}$ for all variants and $\sigma_{i}$ is the standard deviation of $g_{i}$ for all variants.

Variants that satisfied both the pareto optimality constraints and stability constraints were combined to generate double variants. The same pareto optimization and stability constraint method was then used to down-select the final double variants.

The code to generate the *in silico* energies can be found under https://github.com/RedaRawi/iSAPIENS.

#### Antibody expression and purification

Antibody variable heavy chain and light chain sequences were codon optimized, synthesized and cloned into a VRC8400 (CMV/R expression vector)-based IgG1-LS vector as previously described.^30^ The variants were expressed by transient transfection in Expi293 cells (ThermoFisher Scientific, Waltham, MA) using Turbo293 transfection reagent (SPEED BioSystems, Gaithersburg, MD) according to the manufacturer’s recommendation. 50 μg plasmid encoding heavy-chain and 50 μg plasmid encoding light-chain variant genes were mixed with the transfection reagents, added to 100 mL of cells at 2.5 × 10^6^/mL, and incubated in a shaker incubator at 120 rpm, 37°C, 9% CO_2_. At 5 days post-transfection, cell culture supernatant was harvested and purified with a Protein A (GE Healthcare, Chicago, IL) column. The antibody was eluted using IgG Elution Buffer (ThermoFisher Scientific, Waltham, MA) and were brought to neutral pH with 1 M Tris-HCl, pH 8.0. Eluted antibodies were dialyzed against 1xPBS overnight.

#### AlphaLISA characterization of CIS43 variants

AlphaLISA (Perkin-Elmer, Waltham, MA) is a bead-based proximity assay in which singlet oxygen molecules, generated by high energy irradiation of Donor beads, transfer to Acceptor beads, which are within a distance of approximately 200 nm. It is a sensitive high throughput screening assay that does not require washing steps. A cascading series of chemical reactions results in a chemiluminescent signal. The antibody variants expressed in 96-well plates were first quantified by biolayer interferometry and the supernatants were subsequently diluted to 6 nM in transfection media. 5 μL of the IgG supernatants were transferred to an OptiPlate-384 assay plate (white opaque, PerkinElmer, Waltham, MA), mixed with 10μL (10 nM final conc.) of biotinylated peptide probe and 10 μL (10 μg/mL final conc.) of Anti-human IgG (Fc specific; PerkinElmer, Waltham, MA) acceptor beads. After an hour of incubation at RT, non-shaking, 25 μL (40 μg/mL final conc.) of streptavidin donor beads (Perkin-Elmer, Waltham, MA) were added. The plate was then incubated for 30 min at RT in the dark before the AlphaLISA signal was detected using a SpectraMax i3x multi-mode microplate reader (Molecular Devices, San Jose, CA). The antibodies selected from the initial screening of supernatants were expressed and purified in large scale. Purified antibodies were diluted to 10 nM in AlphaLISA buffer (PBS + 0.05% Tween-20 + 0.5 mg/mL BSA) and the AlphaLISA apparent affinity was measured as above.

#### Crystallization and structural analysis

Antibody Fab and peptide 21 (PfCSP residues 101–115) complexes were prepared by mixing 1:2 molar ratio to a concentration of 15 mg/mL. Crystallization conditions were screened in Hampton Research screening kits, Wizard screening kits, Precipitant Synergy screening kits using a mosquito robot. Crystals initially observed from the wells were manually reproduced. The P3-21: P21 complex crystal grew in 5% isopropanol, 2 M lithium sulfate, 0.1 M magnesium sulfate hydrate and 0.1 M sodium acetate trihydrate pH 4.5; the P3-42: P21 complex crystal grew in 0.1 M TRIS hydrochloride pH 8.5, 2.8 M ammonium sulfate, and P3-43:P21 complex crystal grew in 0.1 M TRIS hydrochloride pH 8.5 and 2.25 M ammonium hydrogen phosphate. Crystals were cryoprotected in 20% glycerol and flash-frozen in liquid nitrogen. Data were collected at a temperature of 100 K and a wavelength of 1.00 Å at the SER-CAT beamline ID-22 (Advanced Photon Source, Argonne National Laboratory, Lemont, IL). Diffraction data were processed with the HKL2000 suite. Structure solution was obtained by molecular replacement with Phaser using CIS43 Fab structures (PDB ID: 6B5M) as a search model. Model building was carried out with Coot. Refinement was carried out with Phenix. Ramachandran statistical analysis indicated that the final structures contained no disallowed residues or no more than 0.22% disallowed residues. Data collection and refinement statistics are shown in Table S3.

#### Affinity measurements by BLI

Antibody Fab binding affinity to various ligands were measured using biolayer interferometry on an Octet Red384 instrument (fortéBio) with streptavidin capture biosensors (fortéBio) in solid black tilt-well 96-well plates (Geiger Bio-One). Assays were performed with agitation at 30°C. Immobilization of biotinylated PfCSPm, NPDP19, and Pep21, was performed for 60s, followed by a 60s baseline in buffer (PBS + 1% BSA). Association with Fab (serially diluted from 1000 to 62.5 nM) was done for 60s, followed by a dissociation step in buffer for 180s. In all Octet measurements, parallel correction to subtract systematic baseline drift was carried out by subtracting the measurements recorded for a loaded sensor incubated in PBS. Data analysis was carried out using Octet software, version 9.0. Experimental data were fitted globally with a 1:1 Langmuir model of binding for all the antigens except PfCSPm which was fitted with a 2:1 Langmuir model of binding.

#### Antibody expression and purification

Stable cell pools expressing P3-43-LS were generated by MaxCyte electroporation of CHO-DG44 cells with an expression vector containing the dihydrofolate reductase selection marker and the P3-43-LS heavy chain and light chain genes. Following selection with methotrexate, the recovered stable pools were cultured in a 14-day fed-batch during which the cells were cultured in ActiPro medium supplemented with Cell Boost 7a and 7b (Cytiva) to promote cell growth and protein expression.

Cell culture harvest was clarified by centrifugation (3,000 rpm × 30 min), decanted, and sterile filtered across a 0.8/0.2 μm dual-layered PES membrane (Sartorius). P3-43-LS was captured by Protein A chromatography (Tosoh Bioscience) and eluted in 25 mM sodium citrate, pH 3.5. The eluate was titrated to pH 6.0 with 100 mM MES, pH 7.0, sterile filtered, and conditioned to a conductivity of 6.0 mS/cm by addition of 5 M sodium chloride. Flow-through polishing chromatography was then performed using Capto Q resin (Cytiva), equilibrated in 50 mM MES, pH 6.0. The collected product was concentrated to 13 mg/mL and buffer exchanged into 10 mM histidine, 50 mM sodium chloride, pH 6.0 using tangential flow filtration with a 30 kDa PES flat sheet membrane (MilliporeSigma), followed by terminal sterile filtration.

#### *In vivo* challenge with Pb-PfCSP-SPZ

To evaluate the neutralizing capacity of the CIS43 variants against the malaria SPZs, female B6-albino mice were used, as previously described (Wang et al. Immunity). Briefly, mice were treated intravenously via tail vein with 25 μg or 50 μg antibody diluted in sterile filtered 1X PBS (pH 7.4; total volume 200 mL/mouse). Two hours after antibody infusion, mice were challenged with intravenous injection of 2,000 freshly harvested Pb-PfCSP-GFP/Luc-SPZ into the tail vein.

The malaria burden of infection was measured at 40–42 h after challenge to determine extent of liver infection, and at 6 days to assess blood stage infection or parasitemia. Each mouse received 150 mL of D-Luciferin (30 mg/mL) intraperitoneally. Ten minutes after the Luciferin injection, mice were imaged under isoflurane anesthesia, using IVIS Spectrum *in vivo* imaging system (PerkinElmer). Liver burden and parasitemia were quantified by total flux (photons/sec) expressed by Pb-PfCSP-GFP/Luc-SPZ using the manufacturer’s software (Living Image 4.5, PerkinElmer).

### Quantification and statistical analysis

Functional characterization in malaria challenge model was assessed by the uncorrected Dunn test, as implemented by the GraphPad software, where p-values are defined as ≤ 0.05, ∗; ≤0.01, ∗∗; ≤0.001, ∗∗∗; ≤0.0001, ∗∗∗∗. Errors for affinity measurements correspond to the standard error.
