# Supplementary material for: In silico improvement of affinity for highly protective anti-malarial antibodies
Source: iScience. 2025 Jun 14;28(7):112903. doi: 10.1016/j.isci.2025.112903 (PMC12269619; doi:10.1016/j.isci.2025.112903)
Supplement: Document S1. Figures S1–S5 and Tables S1–S3 [file mmc1.pdf]

iScience, Volume *28*

Supplemental information

In silico improvement of affinity for highly protective anti-malarial antibodies

Mateo Reveiz, Prabhanshu Tripathi, Lais Da Silva Pereira, Patience Kerubo Kiyuka, Tracy Liu, Yongping Yang, Baoshan Zhang, Dorra Benmohamed, Brian G. Bonilla, Carl W. Carruthers Jr., Marlon Dillon, Daniel Gowetski, Sven Kratochvil, Gabriella Lagos, Mariah Lofgren, Ivan Loukinov, Shamika Mathis-Torres, Andrew J. Schaub, Elizabeth Scheideman, Arne Schön, Chen-Hsiang Shen, Yevel Flores-Garcia, Fidel Zavala, Facundo D. Batista, Azza H. Idris, Robert A. Seder, Peter D. Kwong, and Reda Rawi

Figure S1. *In silico* antibody improvement pipeline, related to Figure 1
(A) Pareto optimal solutions from *in silico* energies for double mutants based on m42.127 (left) and m43.151 (right) templates, related to Figure 1. (B) Mutation frequency and source of mutation for the heavy chain. Black mutations from previous *in vivo* mouse models. Mouse model mutations contained in the template antibodies m42.127 and m43.151 in green. Mutations identified by the *in silico* pipeline in blue. Mutations identified by both the *in silico* pipeline and mouse models in red. The height of the residues corresponds to the log of the number of antibodies containing the corresponding mutation.

A Pareto optimal solutions from *in silico* energies for double mutants

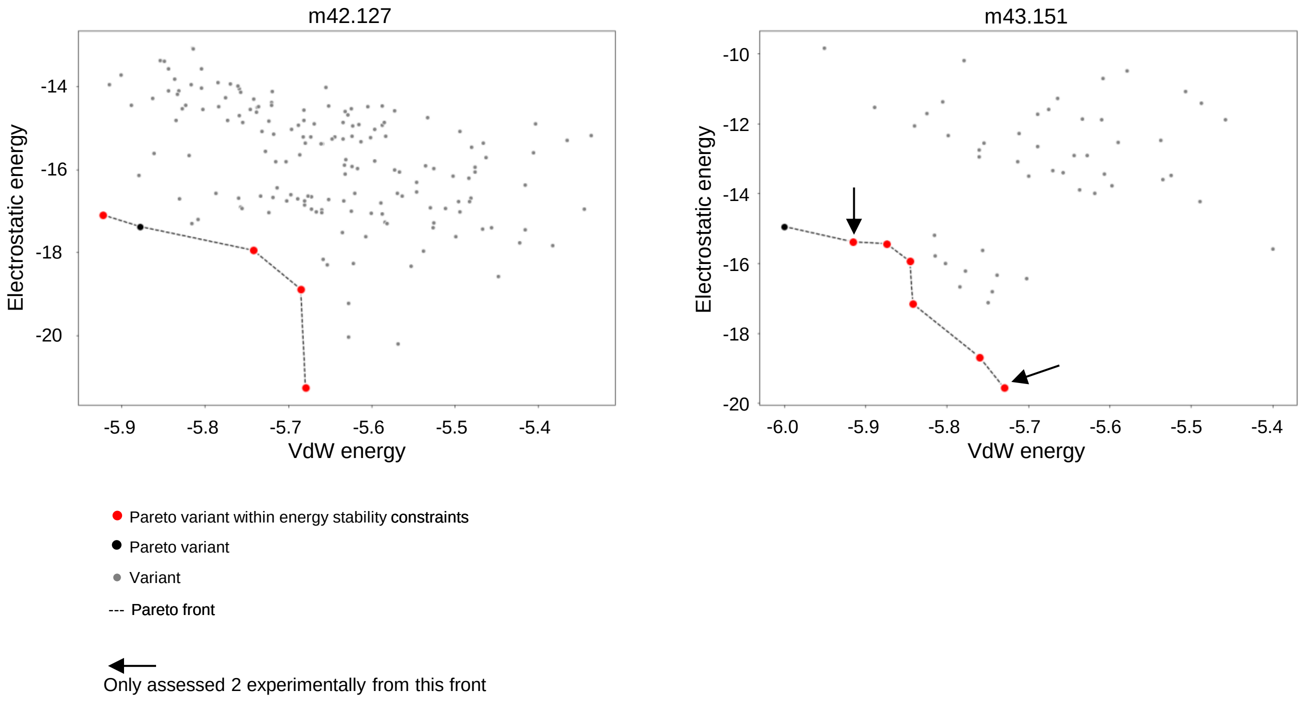

B Heavy chain mutations from previous murine models and *in silico methods*

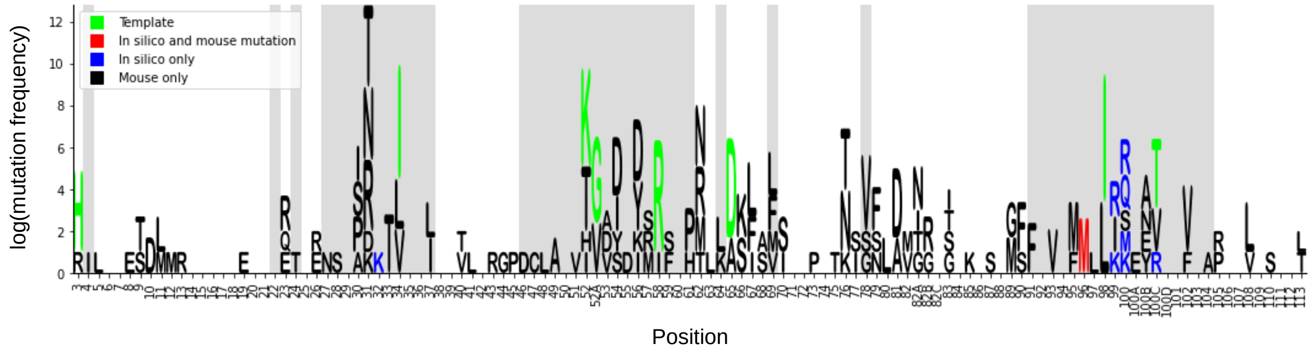

Figure S2. Antigenic screening using AlphaLISA effectively down-selects best antibodies, related to Figure 2
(A) Mean AlphaLISA apparent affinity to NPDP19 measured at 0.6 nM with IgG supernatants.
(B) Mean AlphaLISA apparent affinity to Peptide 21 measured at 1 nM with purified IgGs.
(C) Mean AlphaLISA apparent affinity to Peptide 21 measured at 10 nM with purified IgGs.

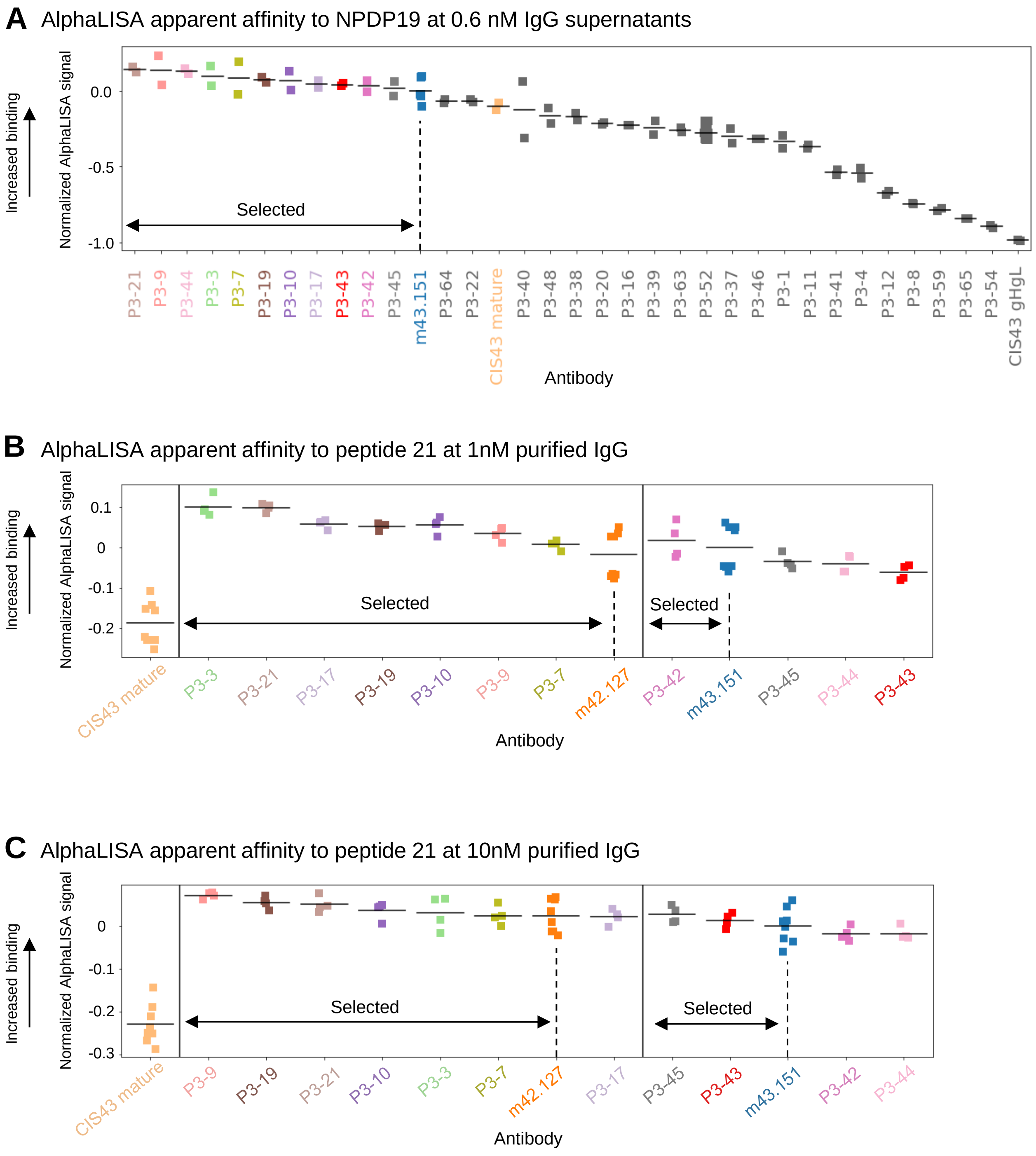

Figure S3. Biolayer interferometry (BLI) affinity and Isothermal titration calorimetry (ITC) of selected CIS43 Antibodies to junctional peptides and *PfCSP*, related to Figure 1

(A) Bio-layer interferometry (BLI) affinity of improved CIS43 variants was determined against NPDP19 and *PfCSPm.* Error bars correspond to the standard error in affinity measurement.

(B) Isothermal titration calorimetry of CSP_SA_mut with various improved CIS43 variants. The affinities and stoichiometry are shown for both KD1 and KD2 in nM.

A BLI affinity

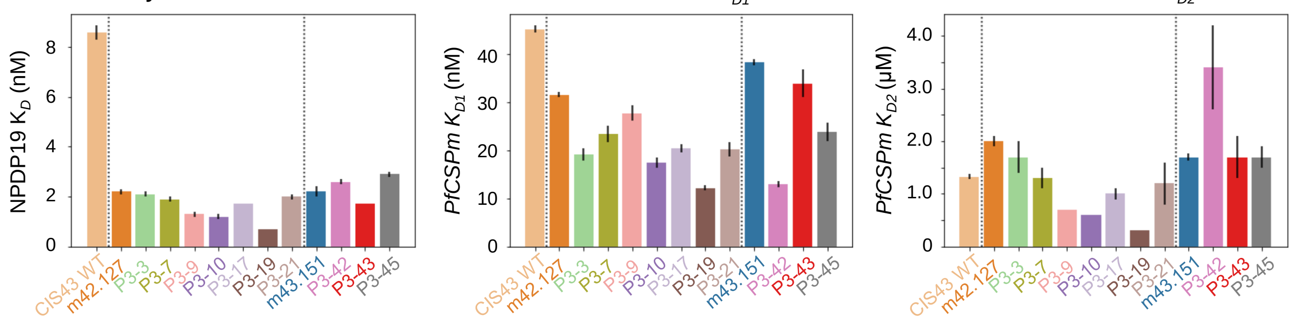

Antibody	*Peptide 21 (nM)*	*NPDP19 (nM)*	*PfCSPm K_{D1} (nM)*	*PfCSPm K_{D2} (μM)*
*CIS43 mature**	61.4 ± 0.3	8.6 ± 0.3	45.2 ± 0.7	1.3 ± 0.0
*CIS43 gHgL**	635.0 ± 48.7	27.2 ± 140	679.0 ± 73	5.0 ± 0.5
*m42.127**	30.2 ± 0.2	2.2 ± 0.1	31.6 ± 0.6	2.0 ± 0.6
P3-3	15.4 ± 0.6	2.1 ± 0.1	19.1 ± 1.3	1.7 ± 0.3
P3-7	24.0 ± 1.0	1.9 ± 0.1	23.4 ± 1.6	1.3 ± 0.2
P3-9	21.8 ± 0.7	1.3 ± 0.1	27.7 ± 1.6	0.7 ± 0.0
P3-10	20.4 ± 0.6	1.2 ± 0.1	17.4 ± 1.0	0.6 ± 0.0
P3-17	17.8 ± 0.5	1.7 ± 0.0	20.4 ± 0.8	1.0 ± 0.1
P3-19	14.6 ± 0.5	0.7 ± 0.0	12.2 ± 0.6	0.3 ± 0.0
P3-21	18.0 ± 0.5	2.0 ± 0.1	20.2 ± 1.5	1.2 ± 0.4
*m43.151**	21.2 ± 0.2	2.2 ± 0.2	38.3 ± 0.7	1.7 ± 0.1
P3-42	14.5 ± 0.3	2.6 ± 0.1	13.0 ± 0.6	3.4 ± 0.8
P3-43	8.9 ± 0.5	1.7 ± 0.0	33.9 ± 2.9	1.7 ± 0.4
P3-45	19.0 ± 0.6	2.9 ± 0.1	23.9 ± 1.9	1.7 ± 0.2

* The affinity data for these antibodies is from Kratochvil et al, 2021, *Immunity*.

B Isothermal titration calorimetry (ITC)

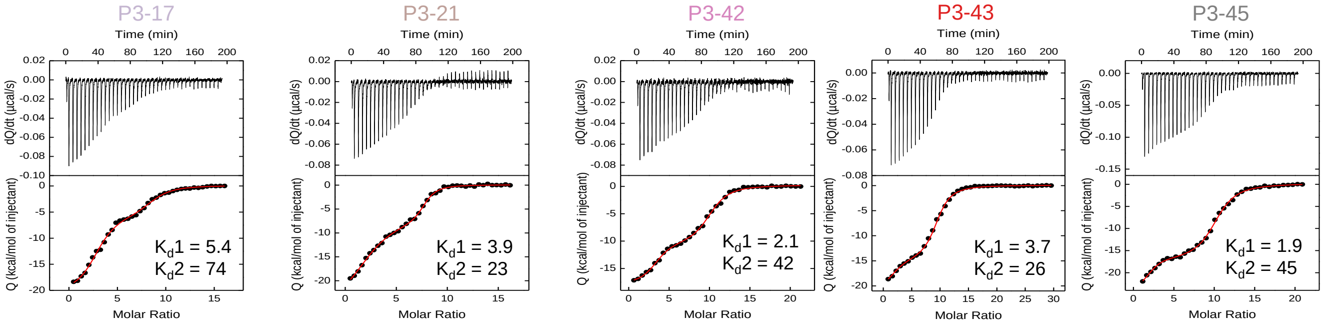

Antibody	Kd1 (nM)	ΔG1 (kcal/mol)	ΔH1 (kcal/mol)	-TΔS1 (kcal/mol)	N1	Kd2 (nM)	ΔG2 (kcal/mol)	ΔH2 (kcal/mol)	-TΔS2 (kcal/mol)	N2
P3-17	5.4	-11.3	-21.1	+9.8	2.8	74	-9.7	-5.8	-3.9	5.7
P3-21	3.9	-11.5	-26.4	+14.9	2.2	23	-10.4	-7.2	-3.2	5.4
P3-42	2.1	-11.8	-18.4	+6.6	3.3	42	-10.1	-10.7	+0.6	6.7
P3-43	3.7	-11.5	-22.4	+10.9	1.9	26	-10.4	-14.7	+4.3	7.4
P3-45	1.9	-11.9	-24.5	+12.6	1.9	45	-10.0	-17.3	+7.3	8.2

Figure S4. *In silico* energies and crystal structure of P3-42 in complex with peptide 21 depict additional atomic interactions potentially explaining increase in affinity, related to Figure 4

(A) *In silico pairwise* energy analysis. For each pair of residues between peptide 21 (Y-axis) and the antibody (X-axis), the total interface energy for the antibody is subtracted from the corresponding template antibody. Lower values indicate more favorable interaction energies. In the bar plots below, values are summed across the peptide 21 positions to further examine the CDRH3 region.

(B) Crystal structures of P3-42 and its template antibody m43.151 are displayed in cartoon illustration. Interatomic interactions from residue 100c in heavy chain and residue 11 in peptide 21 is displayed (bottom right) and compared with that of template m42.151 crystal structure (top right).

A *In silico pairwise* energy analysis

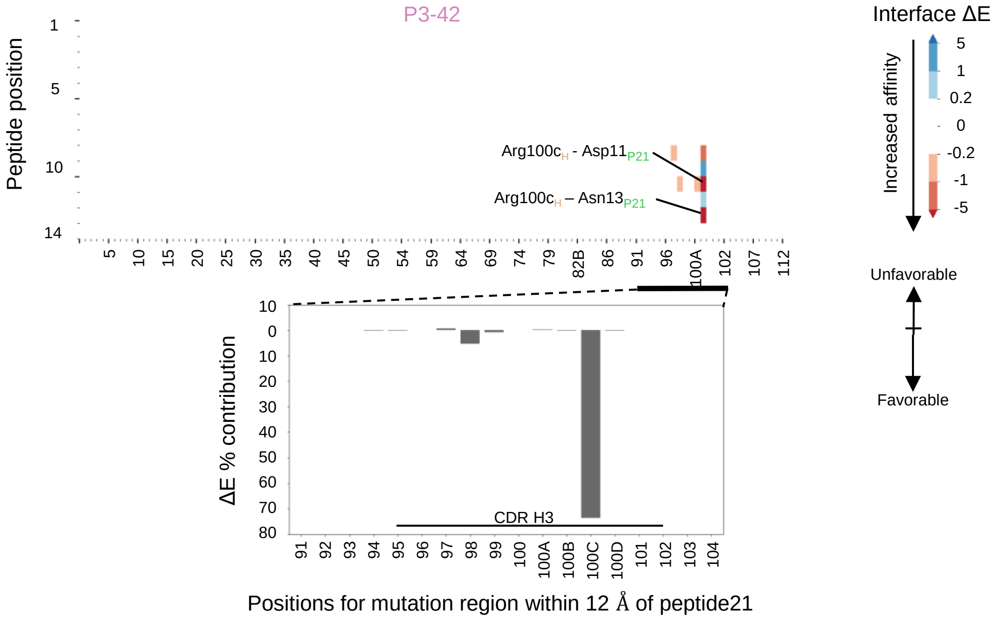

B Crystal structure explains gain in binding affinity by stabilized interaction energies in top antibody P3-42

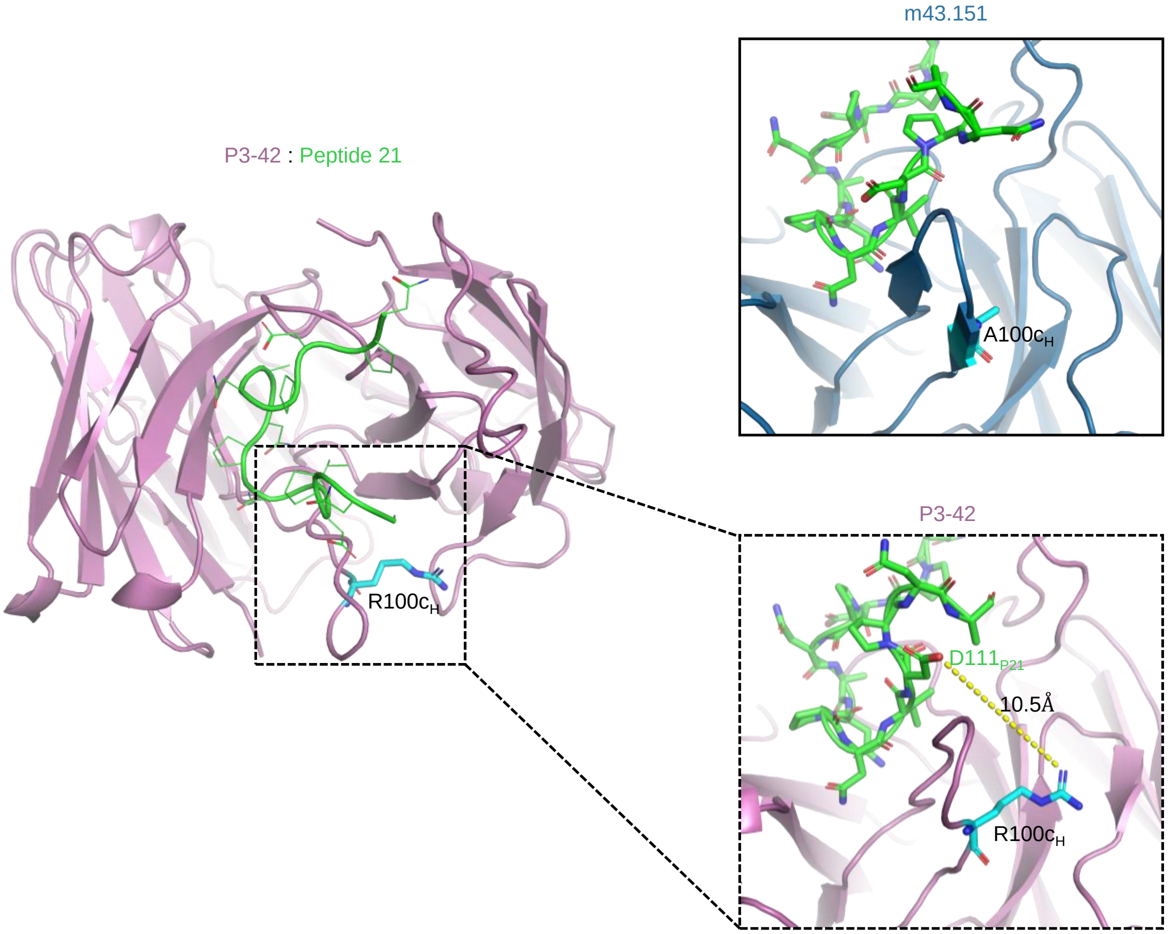

Figure S5. Biolayer interferometry (BLI) analysis of *in silico* designed antibodies to target antigens peptide 21, NPDP19, and PfCSPm
(A, B, and C) BLI curves to peptide 21, NPDP19, and PfCSPm.

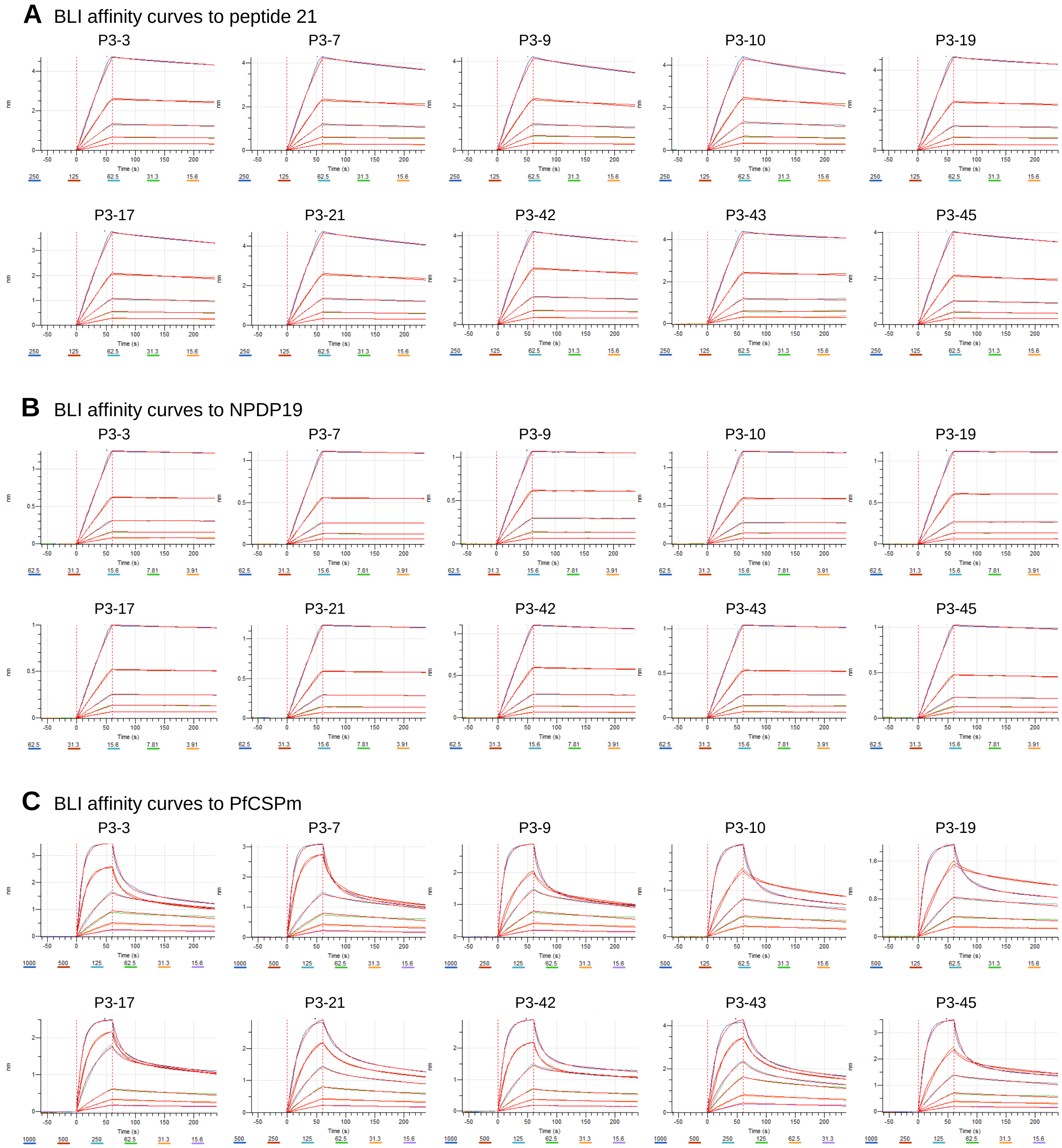

Table S1. *In silico* energies of selected antibodies.

p3_name	backbone	Full name	vdw_int	elec_int	total_int	vdw_HH	elec_HH	total_HH	vdw_LL	elec_LL	total_LL	vdw_HL	elec_HL	total_HL
P3-1	m42_HH28K_13	m42_HH28K_13_TH103R	-5.66603	-17.6253	-23.2915	-149.649	-646.706	-796.355	-154.919	-695.867	-850.787	-12.5092	-10.2183	-22.7276
P3-2	m42_HH28K_13	m42_HH28K_13_AH33W	-5.64712	-13.7243	-19.3714	-149.779	-650.828	-800.608	-154.805	-696.315	-851.12	-12.438	-10.1193	-22.5575
P3-3	m42_HH28K_13	m42_HH28K_13_TH103K	-5.45867	-17.0952	-22.554	-149.383	-645.476	-794.86	-155.089	-696.222	-851.312	-12.4952	-10.1901	-22.6856
P3-4	m42_HH28K_13	m42_HH28K_13_VH101R	-5.58244	-17.0045	-22.587	-149.132	-648.09	-797.223	-154.91	-696.513	-851.423	-12.4553	-10.4127	-22.8685
P3-5	m42_HH28K_13	m42_HH28K_13_NL34W	-5.62732	-14.6412	-20.2687	-148.87	-646.243	-795.114	-154.848	-698.882	-853.731	-12.4751	-10.2372	-22.7127
P3-6	m42_HH28K_13	m42_HH28K_13_SL99H	-5.64214	-14.5703	-20.2126	-148.754	-646.25	-795.004	-154.59	-694.966	-849.556	-12.4358	-9.8828	-22.3187
P3-7	m42_HH28K_13	m42_HH28K_13_TH100M	-5.6454	-14.5622	-20.2076	-149.752	-644.404	-794.157	-154.973	-697.222	-852.195	-12.6447	-10.4906	-23.1356
P3-8	m42_HH28K_13	m42_HH28K_13_VH101K	-5.51827	-16.8772	-22.3955	-148.562	-645.624	-794.187	-154.923	-695.979	-850.904	-12.4118	-10.0323	-22.4443
P3-9	m42_HH28K_13	m42_HH28K_13_PH104M	-5.59082	-14.8877	-20.4786	-148.981	-648.964	-797.945	-154.914	-696.494	-851.409	-12.4999	-10.3349	-22.8352
P3-10	m42_HH28K_13	m42_HH28K_13_PH104Q	-5.51129	-16.0721	-21.5834	-148.941	-648.576	-797.518	-154.935	-696.292	-851.228	-12.5121	-10.2911	-22.8034
P3-11	m42_HH28K_13	m42_HH28K_13_VH101W	-5.60995	-14.5099	-20.1197	-149.308	-646.725	-796.033	-154.921	-696.539	-851.462	-12.4136	-10.2256	-22.6393
P3-12	m42_HH28K_13	m42_HH28K_13_AH97E	-5.50084	-15.2947	-20.7954	-149.96	-645.544	-795.505	-155.219	-696.069	-851.289	-12.5026	-10.5302	-23.0329
P3-13	m42_HH28K_13	m42_HH28K_13_NL34R	-5.38371	-16.191	-21.5747	-148.617	-646.941	-795.559	-154.574	-695.803	-850.377	-12.481	-10.1192	-22.6005
P3-14	m42_HH28K_13	m42_HH28K_13_SL33W	-5.49684	-15.347	-20.8439	-148.614	-645.769	-794.384	-155.233	-696.545	-851.778	-12.4901	-10.0678	-22.5583
P3-15	m42_HH28K_13	m42_HH28K_13_SL99F	-5.57579	-14.627	-20.2029	-148.916	-646.683	-795.6	-154.961	-696.45	-851.411	-12.522	-10.7164	-23.2383
P3-16	m42_HH28K_13	m42_HH28K_13_TH100C	-5.58441	-14.6148	-20.1994	-149.086	-645.993	-795.08	-155.019	-697.17	-852.191	-12.5871	-10.2029	-22.79
P3-17	m42_HH28K_13	m42_HH28K_13_YH32K	-5.44459	-15.6364	-21.0811	-148.39	-646.409	-794.8	-154.888	-696.134	-851.022	-12.5352	-10.3133	-22.8486
P3-18	m42_HH28K_13	m42_HH28K_13_YL38W	-5.57434	-14.8465	-20.4209	-148.919	-646.559	-795.478	-154.851	-696.979	-851.83	-12.4592	-10.4498	-22.9091
P3-19	m42_HH28K_13	m42_HH28K_13_TH103R_PH104R	-5.67921	-21.2676	-26.9468	-149.704	-648.312	-798.016	-154.855	-696.08	-850.936	-12.5306	-10.0552	-22.5861
P3-20	m42_HH28K_13	m42_HH28K_13_TH103R_NL34W	-5.92257	-17.0957	-23.0184	-149.796	-647.319	-797.116	-154.962	-698.22	-853.183	-12.4772	-10.3011	-22.7786
P3-21	m42_HH28K_13	m42_HH28K_13_TH103R_PH104Q	-5.6851	-18.8868	-24.5718	-149.508	-648.353	-797.86	-154.836	-696.426	-851.263	-12.5286	-10.1026	-22.6315
P3-22	m42_HH28K_13	m42_HH28K_13_TH103R_SL33W	-5.74183	-17.9515	-23.6935	-149.594	-647.831	-797.426	-155.42	-696.322	-851.743	-12.5332	-10.0826	-22.6163
-	-	-	-	-	-	-	-	-	-	-	-	-	-	-
P3-37	m43_HH28K_17	m43_HH28K_17_VH101R	-5.739	-16.3869	-22.126	-158.103	-674.793	-832.897	-163.394	-716.407	-879.802	-13.5083	-13.4883	-26.9968
P3-38	m43_HH28K_17	m43_HH28K_17_VH101W	-5.75342	-10.1771	-15.9305	-159.127	-673.339	-832.467	-163.528	-716.255	-879.783	-13.4435	-13.772	-27.2156
P3-39	m43_HH28K_17	m43_HH28K_17_TH100I	-5.70854	-11.2757	-16.9842	-158.257	-671.458	-829.717	-163.385	-717.05	-880.435	-13.7356	-13.4782	-27.2141

P3-40	m43_HH28K_17	m43_HH28K_17_TH103N	-5.64015	-12.9206	-18.5608	-158.839	-672.328	-831.167	-163.801	-716.329	-880.131	-13.5035	-13.6779	-27.1819
P3-41	m43_HH28K_17	m43_HH28K_17_YL38R	-5.64528	-12.0251	-17.6704	-158.327	-671.398	-829.726	-163.103	-718.77	-881.872	-13.5342	-13.882	-27.4165
P3-42	m43_HH28K_17	m43_HH28K_17_AH107R	-5.56919	-12.6155	-18.1849	-159.612	-679.513	-839.126	-163.6	-718.242	-881.842	-13.6544	-13.2961	-26.9506
P3-43	m43_HH28K_17	m43_HH28K_17_PH104K	-5.53911	-12.8779	-18.4169	-157.847	-675.123	-832.971	-163.52	-715.822	-879.343	-13.4613	-13.8786	-27.34
P3-44	m43_HH28K_17	m43_HH28K_17_PH104R	-5.51088	-13.3036	-18.8145	-158.117	-676.104	-834.22	-163.23	-716.388	-879.62	-13.5121	-13.3527	-26.8652
P3-45	m43_HH28K_17	m43_HH28K_17_TH100M	-5.67664	-11.2725	-16.9492	-159.512	-671.162	-830.675	-163.807	-716.542	-880.35	-13.6448	-14.0293	-27.6742
P3-46	m43_HH28K_17	m43_HH28K_17_TH100Q	-5.65571	-11.7839	-17.4397	-158.732	-670.941	-829.673	-163.23	-715.523	-878.754	-13.594	-14.6332	-28.2274
P3-47	m43_HH28K_17	m43_HH28K_17_YH32R	-5.47616	-13.686	-19.1623	-157.9	-671.024	-828.925	-163.371	-716.158	-879.529	-13.5101	-13.9605	-27.4707
P3-48	m43_HH28K_17	m43_HH28K_17_VH101R_PH104R	-5.72961	-19.5615	-25.2911	-158.14	-675.257	-833.397	-163.053	-716.349	-879.402	-13.5224	-13.5951	-27.1177
P3-52	m43_HH28K_17	m43_HH28K_17_TH100I_YH32R	-5.91526	-15.3889	-21.3043	-158.267	-669.191	-827.458	-163.345	-716.285	-879.632	-13.7151	-13.8333	-27.5487
P3-64	m43_HH28K_17	m43_HH28K_17_TH100M_YH32R	-5.87395	-15.4417	-21.3157	-158.671	-670.09	-828.762	-163.271	-715.259	-878.53	-13.694	-14.257	-27.9513
P3-65	m43_HH28K_17	m43_HH28K_17_VH101R_YH32R	-5.84161	-17.1611	-23.0028	-157.925	-670.104	-828.029	-163.483	-716.297	-879.781	-13.5102	-13.6681	-27.1787

Table S2. Variants summary and data availability.

Name	Full Name Kabat	Full name	Backbone	# mutations	Ordered	Expressed	AlphaLISA Supernatants	AlphaLISA Purified	BLI	Liver Burden @ 50ug	Liver Burden @ 25ug	Crystal Struct
P3-1	m42.127_TH99R	m42_HH28K_13_TH103R	m42.127	Single	Yes	Yes	Yes	No	No	No	No	No
P3-2	m42.127_AH33W	m42_HH28K_13_AH33W	m42.127	Single	Yes	No	No	No	No	No	No	No
P3-3	m42.127_TH99K	m42_HH28K_13_TH103K	m42.127	Single	Yes	Yes	Yes	Yes	Yes	Yes	No	No
P3-4	m42.127_VH97R	m42_HH28K_13_VH101R	m42.127	Single	Yes	Yes	Yes	No	No	No	No	No
P3-5	m42.127_NL28W	m42_HH28K_13_NL34W	m42.127	Single	Yes	No	No	No	No	No	No	No
P3-6	m42.127_SL93H	m42_HH28K_13_SL99H	m42.127	Single	Yes	No	No	No	No	No	No	No
P3-7	m42.127_TH96M	m42_HH28K_13_TH100M	m42.127	Single	Yes	Yes	Yes	Yes	Yes	Yes	No	No
P3-8	m42.127_VH97K	m42_HH28K_13_VH101K	m42.127	Single	Yes	Yes	Yes	No	No	No	No	No
P3-9	m42.127_PH100M	m42_HH28K_13_PH104M	m42.127	Single	Yes	Yes	Yes	Yes	Yes	Yes	No	No
P3-10	m42.127_PH100Q	m42_HH28K_13_PH104Q	m42.127	Single	Yes	Yes	Yes	Yes	Yes	Yes	No	No
P3-11	m42.127_VH97W	m42_HH28K_13_VH101W	m42.127	Single	Yes	Yes	Yes	No	No	No	No	No
P3-12	m42.127_AH93E	m42_HH28K_13_AH97E	m42.127	Single	Yes	Yes	Yes	No	No	No	No	No
P3-13	m42.127_NL28R	m42_HH28K_13_NL34R	m42.127	Single	Yes	No	No	No	No	No	No	No
P3-14	m42.127_SL27fW	m42_HH28K_13_SL33W	m42.127	Single	Yes	No	No	No	No	No	No	No
P3-15	m42.127_SL93F	m42_HH28K_13_SL99F	m42.127	Single	Yes	No	No	No	No	No	No	No
P3-16	m42.127_TH96C	m42_HH28K_13_TH100C	m42.127	Single	Yes	Yes	Yes	No	No	No	No	No
P3-17	m42.127_YH32K	m42_HH28K_13_YH32K	m42.127	Single	Yes	Yes	Yes	Yes	Yes	Yes	No	No
P3-18	m42.127_YL32W	m42_HH28K_13_YL38W	m42.127	Single	Yes	No	No	No	No	No	No	No
P3-19	m42.127_TH99R_PH100R	m42_HH28K_13_TH103R_PH104R	m42.127	Double	Yes	Yes	Yes	Yes	Yes	Yes	No	No
P3-20	m42.127_TH99R_NL28W	m42_HH28K_13_TH103R_NL34W	m42.127	Double	Yes	Yes	Yes	No	No	No	No	No
P3-21	m42.127_TH99R_PH100Q	m42_HH28K_13_TH103R_PH104Q	m42.127	Double	Yes	Yes	Yes	Yes	Yes	Yes	Yes	Yes
P3-22	m42.127_TH99R_SL27fW	m42_HH28K_13_TH103R_SL33W	m42.127	Double	Yes	Yes	Yes	No	No	No	No	No
P3-37	m43.151_VH97R	m43_HH28K_17_VH101R	m43.151	Single	Yes	Yes	Yes	No	No	No	No	No
P3-38	m43.151_VH97W	m43_HH28K_17_VH101W	m43.151	Single	Yes	Yes	Yes	No	No	No	No	No
P3-39	m43.151_TH96I	m43_HH28K_17_TH100I	m43.151	Single	Yes	Yes	Yes	No	No	No	No	No
P3-40	m43.151_TH99N	m43_HH28K_17_TH103N	m43.151	Single	Yes	Yes	Yes	No	No	No	No	No
P3-41	m43.151_YL32R	m43_HH28K_17_YL38R	m43.151	Single	Yes	Yes	Yes	No	No	No	No	No
P3-42	m43.151_AH100cR	m43_HH28K_17_AH107R	m43.151	Single	Yes	Yes	Yes	Yes	Yes	Yes	No	Yes

P3-43	m43.151_PH100K	m43_HH28K_17_PH104K	m43.151	Single	Yes	Yes	Yes	Yes	Yes	Yes	Yes	No
P3-44	m43.151_PH100R	m43_HH28K_17_PH104R	m43.151	Single	Yes	Yes	Yes	Yes	No	No	No	No
P3-45	m43.151_TH96M	m43_HH28K_17_TH100M	m43.151	Single	Yes	Yes	Yes	Yes	Yes	Yes	No	No
P3-46	m43.151_TH96Q	m43_HH28K_17_TH100Q	m43.151	Single	Yes	Yes	Yes	No	No	No	No	No
P3-47	m43.151_YH32R	m43_HH28K_17_YH32R	m43.151	Single	Yes	No	No	No	No	No	No	No
P3-48	m43.151_VH97R_PH100R	m43_HH28K_17_VH101R_PH104R	m43.151	Double	Yes	Yes	Yes	No	No	No	No	No
P3-52	m43.151_TH96I_YH32R	m43_HH28K_17_TH100I_YH32R	m43.151	Double	Yes	Yes	Yes	No	No	No	No	No
P3-64	m43.151_TH96M_YH32R	m43_HH28K_17_TH100M_YH32R	m43.151	Double	No	No	No	No	No	No	No	No
P3-65	m43.151_VH97R_YH32R	m43_HH28K_17_VH101R_YH32R	m43.151	Double	No	No	No	No	No	No	No	No

Table S3: X-ray crystallography data collection and refinement statistics.

	P3-21 : Pep21	P3-42 : Pep21	P3-43 : Pep21
PDB accession code	*7UFN*	*7UFO*	*7UFQ*
**Data collection**			
Wavelength	1	1	1
Resolution range (Å)	51.82 - 2.20 (2.28 - 2.20)	45.52 – 1.8 (1.86-1.80)	43.52 – 2.30 (2.38 – 2.30)
Space group	P 2_1	C 2_1	P 2_1
Cell dimensions			
a, b, c (Å)	36.7 124.0 103.6	94.5 60.7 86.5	74.5 61.7 89.6
a, b, g (°)	90.0, 90.1, 90.0	90.0, 106.1, 90.0	90, 103.7, 90.0
Unique reflections	46287 (4700)	42986 (4118)	34697 (3381)
Multiplicity	3.6 (3.6)	3.5 (3.2)	3.4 (3.6)
Completeness (%)	99.8 (99.9)	98.2 (94.3)	97.45 (96.88)
I / σI	5.7 (2.0)	7.5 (1.9)	8.92 (2.10)
Wilson *B*-factor	25.21	15.59	32.07
R_{merge}	0.159 (0.562)	0.099 (0.572)	0.203 (1.017)
$CC_{1/2}$	0.98 (0.79)	0.99 (0.76)	0.984 (0.587)
**Refinement**			
Reflections used in refinement	46921 (4700)	42944 (4104)	34476 (3381)
Reflections used for R-free	2448 (248)	2208 (213)	1702 (175)
R_{work}	0.17 (0.13)	0.19 (0.26)	0.22 (0.27)
R_{free}	0.20 (0.17)	0.22 (0.30)	0.28 (0.31)
Number of non-hydrogen atoms	6947	3811	7037
macromolecules	6891	3456	6916
ligands	25	22	6
solvent	31	333	115
Protein residues	903	455	906
RMS(bonds) (Å)	0.009	0.007	0.010
RMS(angles) (°)	1.21	1.05	1.27
Ramachandran favored (%)	95.29	97.30	95.41
Ramachandran outliers (%)	0.11	0.22	0.0
Average *B*-factor ($Å^2$)	24.74	21.92	33.70
macromolecules	24.56	21.50	33.75
ligands	61.73	26.02	20.00
solvent	35.60	25.95	31.21
